# Molecular basis of stepwise cyclic tetra-adenylate cleavage by the type III CRISPR ring nuclease Crn1/Sso2081

**DOI:** 10.1093/nar/gkad101

**Published:** 2023-02-20

**Authors:** Liyang Du, Danping Zhang, Zhipu Luo, Zhonghui Lin

**Affiliations:** College of Chemistry, Fuzhou University, Fuzhou 350108, China; College of Chemistry, Fuzhou University, Fuzhou 350108, China; Institute of Molecular Enzymology, School of Biology and Basic Medical Sciences, Suzhou Medical College of Soochow University, Suzhou, China; College of Chemistry, Fuzhou University, Fuzhou 350108, China; Key Laboratory of Marine Enzyme Engineering, Fuzhou University, Fuzhou, China

## Abstract

The cyclic oligoadenylates (cOAs) act as second messengers of the type III CRISPR immunity system through activating the auxiliary nucleases for indiscriminate RNA degradation. The cOA-degrading nucleases (ring nucleases) provide an ‘off-switch’ regulation of the signaling, thereby preventing cell dormancy or cell death. Here, we describe the crystal structures of the founding member of CRISPR-associated ring nuclease 1 (Crn1) Sso2081 from *Saccharolobus solfataricus*, alone, bound to phosphate ions or cA_4_ in both pre-cleavage and cleavage intermediate states. These structures together with biochemical characterizations establish the molecular basis of cA_4_ recognition and catalysis by Sso2081. The conformational changes in the C-terminal helical insert upon the binding of phosphate ions or cA_4_ reveal a gate-locking mechanism for ligand binding. The critical residues and motifs identified in this study provide a new insight to distinguish between cOA-degrading and -nondegrading CARF domain-containing proteins.

## INTRODUCTION

The CRISPR system provides adaptive immunity against mobile genetic elements in bacteria and archaea (1–3). Upon the invasion of foreign DNA and/or RNA, the surveillance systems are activated to degrade the invaders through the ribonucleoprotein (RNP) complexes (4–7). According to the composition of RNP complexes, CRISPR systems can be divided into two classes (8,9). The class 1 systems (types I, III and IV) consist of multi-subunit RNPs (10), whereas the class 2 systems (types II, V and VI) possess single-subunit RNPs (11,12).

The Type III CRISPR systems are featured by the presence of Cas10 signature protein (also named as Csm1 or Cmr2), which contains an HD nuclease domain for ssDNA cleavage and a cyclase domain for cyclic oligoadenylate (cOA) synthesis (13–17). The recognition of a target RNA by RNP complex stimulates the cyclase domain to synthesize cOA molecules. These cOA molecules (typically ranging 3–6 AMPs), as second messengers, in turn allosterically activate the CRISPR ancillary nucleases such as Csx1/Csm6 (18,19), Can1/Can2 (CRISPR ancillary nuclease 1/2) (20,21), and Card1 (cyclic-oligoadenylate-activated single-stranded ribonuclease and single-stranded deoxyribonuclease 1) (22), resulting in indiscriminate degradation of both foreign and host DNA and / or RNA (23,24). Therefore, although cOA is critical for host immunity against foreign genetic elements, its cellular level must be tightly controlled so as to avoid cell dormancy or cell death (25).

Recently, a group of CRISPR-associated Rossman-fold (CARF) domain containing proteins, termed the ring nucleases, have been described to cleave cOA molecules in a metal-independent mechanism (26–32). Based on the functionality, these ring nucleases can be divided into two major categories: (i) the standalone ring nucleases that are specific for cOA degradation, such as the *S. solfataricus* (Sso) Sso2081 and Sso1393 (26), *S. islandicus* (Sis) Sis0811 (27) and Sis0455 (33) (Crn1), the anti-CRISPR (Acr) III-1 (Crn2) (28), and Csx3 (Crn3) (29); (ii) the self-limiting cOA-dependent ribonucleases like Csm6 from *T. onnurineus* (30), *T. thermophilus* (31), *E. italicus* (32) and *S. thermophilus* (34), which also consist of a HEPN (higher eukaryotes and prokaryotes nucleotide) domain for DNA and/or RNA degradation.

Sso2081 is the founding member of ring nuclease family identified from *S. solfataricus* (26). It has been shown that Sso2081 could convert cA_4_ into 5′-OH-ApA-2′,3′-cyclic phosphate (A_2_ > P), and hence inactivate the RNase activity of Csx1 in target RNA clearance (26). These findings represent an important milestone in our understanding of the regulations of CRISPR system, however, the structural mechanisms of cA_4_ recognition and cleavage by Sso2081 remain to be established. In the present work, we have determined the crystal structures of Sso2081, alone, bound to phosphate ions or cA_4_ in both pre-cleavage and transient intermediate states. These structures together with extensive biochemical analyses extend our understanding of the molecular basis of ‘off-switch’ regulation for the CRISPR system.

## MATERIALS AND METHODS

### Oligonucleotides and cloning

cA_4_ (cyclic tetraadenosine monophosphate) was ordered from Biolog Life Science Institute, Bremen, Germany. The Sso2081 cDNA (GenBank ID: 1559988754) was synthesized at GenScript Corporation (Nanjing, China). The cDNA of Sso2081 and its variants were subcloned into a modified pET bacterial expression vector with an N-terminal cleavable His_6_-tag.

### Protein expression and purification

The Sso2081 protein was expressed in *Escherichia coli* Rosetta (DE3) cells. Cells cultured to OD_600_ ∼0.6 were induced with 0.5 mM isopropyl β-d-1- thiogalactopyranoside (IPTG) at 18°C overnight. Then, the cells were harvested, resuspended with lysis buffer (20 mM Tris–HCl pH 8.0, 200 mM NaCl, 10 mM imidazole, 5% glycerol and 0.1% Tween-20), and disrupted by French Pressure (Union Biotech, China). The His_6_-tagged protein in the supernatant was pooled through Ni-NTA resin (Union Biotech, China). The column was subjected to extensive wash with 40 mM imidazole containing lysis buffer, and the protein of Sso2081 was eluted with lysis buffer supplemented with 200 mM imidazole. After removal of His_6_ tag with the home-made preScission protease, the untagged protein was further purified through 15Q anion exchange column (GE Healthcare Life Sciences) and Uniondex 200 pg 16/60 size-exclusion column (Union Biotech, China). The final purified protein in 20 mM Tris–HCl pH 8.0 and 150 mM NaCl was concentrated and stored at –80°C. The proteins of various Sso2081 mutants were expressed and purified similarly.

The selenomethionine (SeMet) labelled protein of Sso2081 was produced as previously described (35). Briefly, the Rosetta (DE3) cells containing pET-Sso2081 plasmid were cultured in Luria-Bertani (LB) media overnight. The cells were collected and washed with M9 minimal media, and further cultured in M9 minimal media at 37°C until the OD_600_ reached about 0.6. Then, the amino acid mixture containing 50 mg/l of leucine, isoleucine and valine, 100 mg/l of phenylalanine, lysine and threonine, and 80 mg/l of SeMet was added to the culture. Protein expression was then induced with 0.5 mM IPTG at 18°C overnight. The SeMet labelled protein was subsequently purified using the same protocol as described above.

### cA_4_ cleavage assay

A cA_4_ cleavage assay was conducted to determine the ring nuclease activities of Sso2081 and its variants. In 50 μl reaction, 40 μM of synthetic cA_4_ was incubated with 2 μM or indicated concentrations of Sso2081 in the cleavage buffer containing 20 mM Tris–HCl pH 8.0 and 50 mM NaCl at 60°C for 30 min or indicated time. At the end of the reaction, 50 μl chloroform-isoamylol (24:1) was added, followed by vortexing for 60 s. Then the samples were centrifuged at 10 000 rpm for 8min. After another round of chloroform-isoamylol extraction, the top aqueous phase containing nucleotides were collected for further analyses on liquid chromatography (LC) and mass spectrometry (MS).

### LC–MS analyses

The cA_4_ and its cleavage products extracted by chloroform-isoamylol were separated using a high performance liquid chromatography (HPLC) system (LC-20A, Shimadzu) equipped with a RX-C18 column (2.1 × 100 mm, 5 μm, Zhongpu Science). After injection of 20 μl sample, the column was eluted with a linear gradient of buffer-B (acetonitrile supplemented with 0.01% TFA) against buffer-A (water with 0.01% TFA) at a flow rate of 0.35 ml/min as follows: 0–2 min, 2–20% B; 2–5 min, 20% B; 5–12 min, 20–48% B; 12–13 min 48–95% B; 13–25 min 95% B; 25–26 min, 95–2% B; 26–35 min, 2% B. The column temperature was set to 40°C and the UV data were recorded with a wavelength of 259 nm. Mass spectra data were acquired in negative-ion mode with scan range of *m/z* 150–1500 on an Agilent 6520 ACURATE-Mass Q-TOF mass spectrometer. The mass spectrometer was operated in full scan and multiple reaction monitoring (MRM) modes. The capillary voltage was 3.5 kV and the temperature was 350°C. Nebulizer pressure was set to 40 psi, and the drying gas flow rate was 10 l/min. Nitrogen was used as nebulizer and auxiliary gas.

### Crystallization and data collection

Crystallizations were performed at 25°C using the hanging-drop vapor diffusion method. Crystallization drops were set up by mixing protein or protein-ligand complex with equal volume of reservoir solutions. Crystals were cryoprotected by the reservoir solutions supplemented with 15–25% glycerol prior to data collection.

The crystals of SeMet-Sso2081 were grown with 20 mg/ml of protein in the reservoir solution consisting of 0.1 M sodium acetate trihydrate pH 4.5 and 25% w/v polyethylene glycol 1500. For the crystallization of Sso2081^Tyr133Phe^ mutant, 17 mg/ml of protein was mixed in reservoir solution containing 0.15 M potassium bromide and 30% w/v polyethylene glycol monomethyl ether 2000. For the crystallization of Sso2081/cA_4_ complex, 16 mg/ml of Sso2081 protein was pre-incubated with cA_4_ at 4°C with a molar ratio of 1:1.5, the crystals were grown with a reservoir solution containing 0.1 M sodium acetate trihydrate pH 5.0, 22% w/v polyethylene glycol monomethyl ether 550 and 5% w/v *n*-dodecyl-β-d-maltoside. For the crystallization of Sso2081^Ser11Ala^/A_4_ > P complex, 16 mg/ml of Sso2081^Ser11Ala^ protein was pre-incubated with cA_4_ at 25°C with a molar ratio of 1:1.5, crystals were obtained using the reservoir solution containing 0.1 M sodium acetate trihydrate, pH 5.0 and 20% w/v polyethylene glycol 1500.

X-ray diffraction data of the crystals of SeMet-Sso2081 and Sso2081^Ser11Ala^/A_4_ > P were collected at beamline of BL02U1 with a wavelength of 0.979 Å, the data of Sso2081/cA_4_ were collected at BL19U1 with a wavelength of 0.978 Å, and the data of Sso2081 Tyr133Phe were collected at BL18U1 with a wavelength of 0.979 Å, at National Facility for Protein Science in Shanghai (NFPS), at Shanghai Synchrotron Radiation Facility (SSRF). Diffracting data were processed with XDS (36) and HKL2000 softwares (37).

### Structure determination and refinement

The initial phase for structure determination was obtained by the selenium single anomalous dispersion (SAD) method. The crystal of SeMet-Sso2081 diffracted to 2.70 Å resolution and exhibited the symmetry of the space group *P*2_1_ with cell dimensions of *a* = 67.03 Å, *b* = 38.38 Å and *c* = 69.80 Å; α = 90.00º, β = 106.41º and γ = 90.00º. Using the data truncated to 2.70 Å, eight possible selenium sites were located and refined with the Autosol program in the PHENIX package (38), resulting in an overall figure of merit of 0.31. The resulting electron density map was used to construct an initial model with the AutoBuild program in PHENIX (39).

The structures of Sso2081^Tyr133Phe^, Sso2081/cA_4_ and Sso2081^Ser11Ala^/A_4_ > P were solved by molecular replacement with the program Phaser-MR of PHENIX (40), using the structure of SeMet-Sso2081/phosphate as template. The resulting solution was used to construct the initial model with the program AutoBuild in PHENIX (39). Iterative model building and refinement were carried out with COOT (41), PHENIX (38) and REFMAC5 (42). The final models were validated with the program MolProbity in PHENIX (38). Data collection and refinement statistics were summarized in Table 1. All structural figures in this study were generated with the PyMOL program (http://www.pymol.org/).

**Table 1.** Data collection and structure refinement statistics

	SeMet-Sso2081/phosphate	Sso2081^Tyr133Phe^	Sso2081/cA_4_	Sso2081^Ser11Ala^/cA_4_> P
**Data collection**				
Space group	*P*2_1_	*P2*_1_	*P*2_1_	*P*2_1_
Cell dimensions				
*a*, *b*, *c* (Å)	67.03, 38.38, 69.80	43.75, 95.25, 45.84	67.05, 38.84, 72.97	67.51, 39.14, 74.09
α, β, γ (º)	90, 106.4, 90	90, 107.8, 90	90, 105.8, 90	90, 105.2, 90
Resolution (Å)	40.95–2.70	50.00–2.00	50.00–3.11	50.00–2.50
	(2.80–2.70)^a^	(2.05–2.00)	(3.22–3.11)	(2.59–2.50)
*R*_merge_ (%)	6.7 (74.8)	8.6 (118.3)	7.0 (78.6)	4.3 (85.1)
(*I*)/σ*(I*)	10.9 (1.5)	11.6 (1.6)	22.8 (1.9)	32.2 (1.9)
Completeness (%)	93.9 (95.6)	97.6 (98.3)	99.57 (97.89)	99.9 (92.6)
Redundancy	5.3 (5.3)	4.7 (4.9)	3.7 (3.5)	6.4 (5.8)
**Refinement**				
Resolution (Å)	40.95–2.70	47.63–2.00	28.56–3.11	28.91–2.50
No. reflections	8615	22937	6382	12407
*R*_work_/*R*_free_	0.263/0.288	0.224/0.256	0.238/0.258	0.258/0.279
No. atoms	2710	2911	2937	2956
Macromolecules	2641	2820	2816	2822
Ligand/ion	66	0	88	88
Water	3	62	1	6
*B*-factors	124.3	51.1	139.9	117.7
Macromolecules	124.3	51.2	140.9	118.6
Ligand/ion	126.7	0	109.4	90.7
Water	67.3	47.5	42.7	83.4
R.m.s. deviations				
Bond lengths (Å)	0.010	0.010	0.010	0.010
Bond angles (°)	1.80	1.600	1.71	1.76
Ramachandran plot (%)				
Favored/allowed/ disallowed	94.41/5.59/0.00	97.46/2.64/0.00	93.58/6.42/0.00	96.09/3.91/0.00

^a^Values in parentheses are for highest-resolution shell.

### Microscale thermophoresis (MST) binding assay

The binding affinities of Sso2081 and its variants with cA_4_ were evaluated by the microscale thermophoresis (MST) binding assay, using the instrument of NanoTemper Monolith NT.115 (NanoTemper Technologies, München, Germany). Proteins were fluorescently labelled with the RED-NHS dye (MO-L011, NanoTemper Technologies) and the free dyes were removed with phosphate buffered saline pH 7.4 supplemented with 0.05% tween-20 (PBST). Then, 50 nM of the labelled protein was incubated with varying concentrations of cA_4_ at room temperature for 15 min in PBST buffer. The samples were loaded into the NanoTemper capillaries (MO-K022, NanoTemper Technologies) and measured at 25°C using 60–100% LED power and medium MST power. All experiments were performed in triplicate and data were analyzed using NanoTemper analysis software.

## RESULTS

### Crystal structure of sso2081 in complex with phosphate ions

The full length Sso2081 proteins (aa.1–178) were expressed and purified from bacterial cells. We first tested whether the recombinant protein of Sso2081 was active in cA_4_ cleavage by high-performance liquid chromatography-mass spectrometry (HPLC-MS) analyses. The recombinant Sso2081 could cleave synthetic cA_4_ in both time- and concentration- dependent manners (Supplementary Figure S1), yielding a single-turnover rate constant of ∼0.83 min^−1^, which is comparable to the previous results (0.23 min^−1^) (26). In agreement with the previous findings (26), Sso2081 could convert cA_4_ into two major species, the linear intermediate 5′-OH-ApApApA-2′,3′-cyclic phosphate (A_4_ > P) and the final product A_2_ > P (Supplementary Figure S1).

We next crystallized the native protein of Sso2081. Molecular replacements using the structures of Sso1393 (PDB: 3QYF) and Sis0811 (PDB: 7PQ2) did not result in a reasonable solution. We then tried to determine the structure by single-wavelength anomalous dispersion (SAD) method. The selenomethionine (SeMet)-labeled Sso2081 crystals diffracted to a minimum Bragg spacing of 2.70 Å resolution, and the structure was solved using the Autosol program in the PHENIX package (38). The final refined model contains two molecules in the asymmetric unit with good geometry. Data collection and refinement parameters were summarized in Table 1.

**Figure 1.**
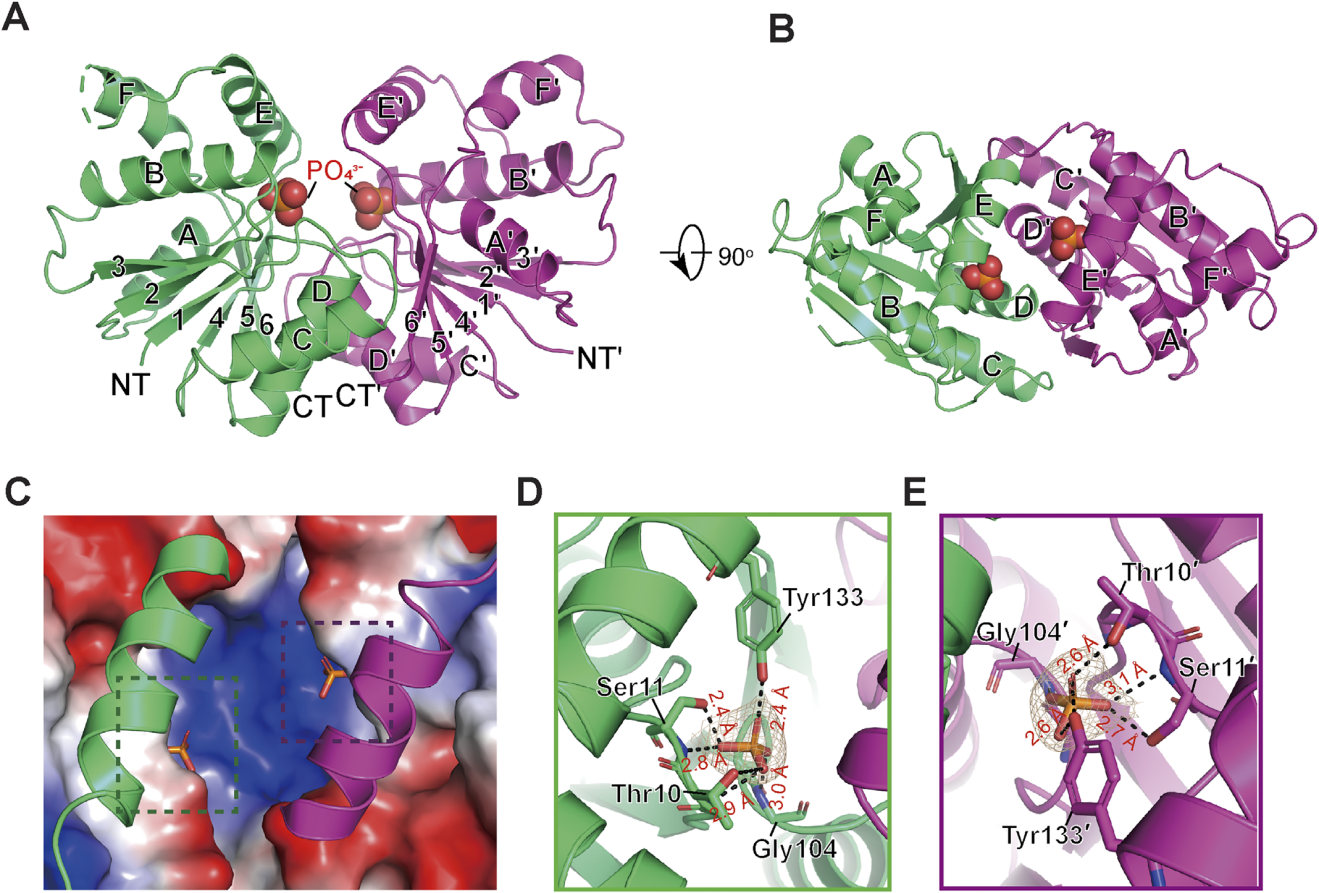
Crystal structure of the Sso2081 in complex with phosphate ions. (**A**) The overall structure of Sso2081/phosphate complex. The structure is shown in cartoon representation and the bound phosphate ions are in spheres. The two homo-monomers are colored in green and magenta. The secondary structural elements are labeled. The N and C termini are indicated as NT and CT. (**B**) Top-view of the Sso2081/phosphate structure. (**C**) The surface drawing of Sso2081 active sites bound to phosphate ions. The helical inserts are shown in cartoon. The surface is colored according to the electrostatic potential (blue, positive; red, negative; white, neutral). (**D, E**) The phosphate ion-binding sites in Sso2081. The 2Fo − Fc electron density map of the bound phosphates shown at 2.0 σ. Key interacting residues are shown as sticks and labeled. Dashed lines indicate hydrogen bonds. All structural figures in this study, unless otherwise indicated, use the same color and labeling schemes.

So far, the CARF domains of all solved structures have been shown to form dimers (43). In agreement with this, the structure of Sso2081 reveals a butterfly-shaped homodimer in a two-fold symmetry (Figure 1A and B). In each monomer, there are six β-strands sandwiched by six α-helices with four on one side and two on the other side, forming a canonical Rossman fold. The dimerization of the two monomers mainly involves helices C, D and β6, which together generate a buried surface of about 1198.7 Å^2^. A structural homology search with the DALI server (44) revealed that Sso2081 is structurally related to the CARF domains of SthCan2 (PDB: 7BDV, RMSD = 2.2 Å) (21) and TsuCard1 (PDB: 6WXX, RMSD = 2.6 Å) (Supplementary Figure S2) (22). The most prominent feature of Sso2081 is the presence of the C-terminal helical insert (αE and αF), which functions as a lid covering the catalytic center (Figure 1C).

Like many other ring nucleases, the catalytic pocket of Sso2081 is located above the dimer interface, featured by a central positively charged region (Figure 1C). This basic patch likely binds the phosphodiester backbone of cA_4_ substrates as revealed by the previous cA_4_-bound CARF domains (21,27). Notably, two phosphate ions, presumably from the protein purification process, bind two symmetrical catalytic pockets with unambiguous electron density (Figure 1D and E). Each phosphate binds one Sso2081 monomer and forms multiple hydrogen bonds with residues Thr10, Ser11 and Gly104. Of note, Tyr133 from the helical insert is also involved in phosphate binding (Figure 1D and E). These residues are absolutely conserved among Sso2081 homologs from different species (Supplementary Figure S3).

### Crystal structure of sso2081 in its apo form

**Figure 2.**
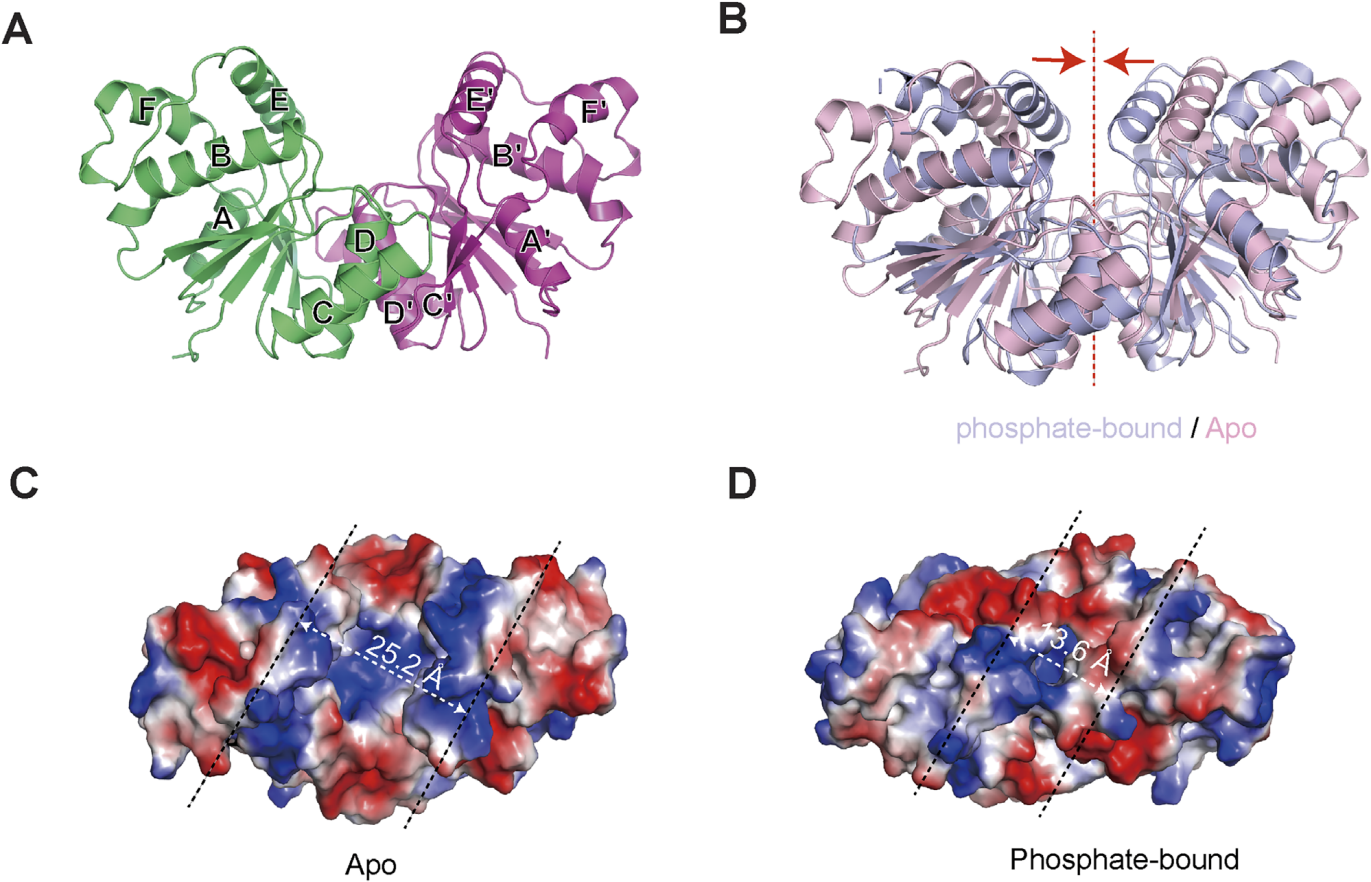
Structure of Sso2081 in its apo form. (**A**) Cartoon diagram of the crystal structure of Sso2081^Tyr133Phe^. (**B**) Structural comparison between the apo (light pink) and phosphate-bound (light blue) forms of Sso2081. Arrows indicate the movement of helical inserts upon phosphate binding. Dash line indicates the dimeric axis. (**C**, **D**) The surface presentation of the active sites of Sso2081 at apo (C) and phosphate-bound (D) states. The surface is colored according to the electrostatic potential (blue, positive; red, negative; white, neutral). The distance between the main-chain backbones of helices αE and αE′ is indicated.

Since the active sites of Sso2081/phosphate complex are already occupied by the phosphate ions, it may not represent the apo form. To rule out this possibility, we next generated a Tyr133Phe mutation in order to abrogate the interaction of the helical insert with phosphate ion. We then crystallized the Sso2081^Tyr133Phe^ protein and determined its structure at a resolution of 2.0 Å (Table 1). As expected, no phosphates were found in the structure of Sso2081^Tyr133Phe^ (Figure 2A and B). Remarkably, a structural comparison between the apo and phosphate-bound Sso2081 revealed major conformational changes in the helical insert, with a root-mean-square deviation (RMSD) value of 4.2 Å (Figure 2B). In the absence of phosphate ions, the helices αE of the two monomers move apart from each other with a distance of about 25.2 Å (Figure 2C), which is only about 13.6 Å in the phosphate-bound structure (Figure 2D). Thus, the structure of Sso2081^Tyr133Phe^ can be the true apo form, in which the active sites are wide-open for substrate access.

### Mechanism of cA_4_ recognition by sso2081

**Figure 3.**
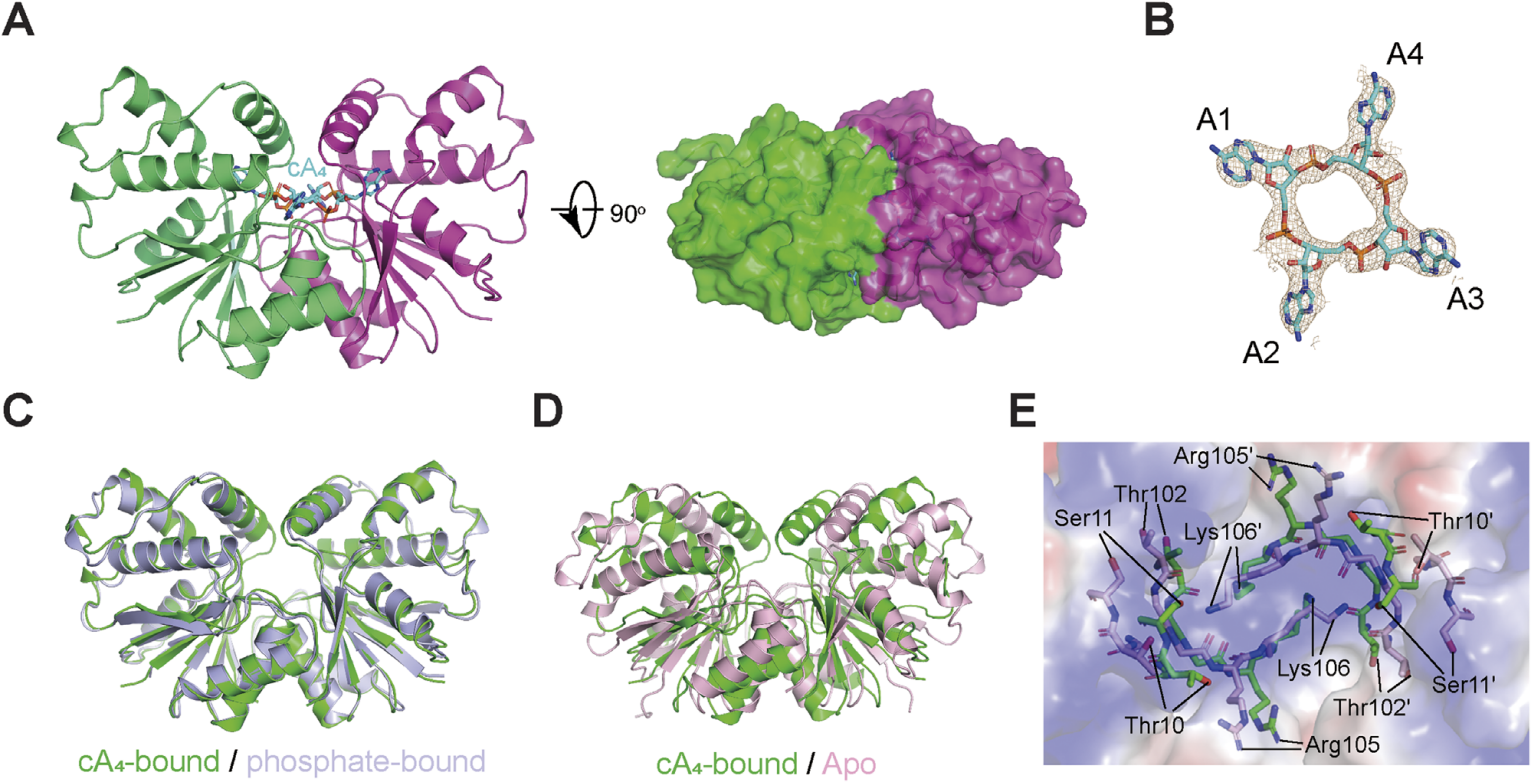
Structure of Sso2081 in complex with cA_4_. (**A**) Cartoon diagram of the crystal structure of Sso2081/cA_4_ complex from side (left panel) and top (right panel, overlaid with a 20% transparent surface) views. cA_4_ is shown as cyan stick. (**B**) The 2Fo – Fc electron density map (contoured at 1.0 σ) of cA_4_ in the crystal structure. (**C**) Structural comparison between the cA_4_- (green) and phosphate (light blue)-bound Sso2081. (**D**) Structural comparison between the apo (light pink) and cA_4_-bound (green) Sso2081. (**E**) Local rearrangements in the active site of Sso2081 upon cA_4_ binding. The key residues for apo and cA_4_-bound Sso2081 are shown in light pink and green sticks, overlaid with a 60% transparent surface of the active site of apo-Sso2081.

To investigate the mechanism of substrate recognition by Sso2081, we next determined the crystal structure of Sso2081 in complex with cA_4_ (Table 1). In the structure, cA_4_ adopts a two-fold symmetry and is completely buried into the catalytic pocket (Figure 3A). The four phosphodiester bonds do not appear to be cleaved as evidenced by the continuous electron density along the ring (Figure 3B), implying that the structure was determined at the pre-cleavage state. The overall structure of Sso2081 is virtually identical to that of phosphate-bound Sso2081 (RMSD ∼ 0.73 Å), but significantly different from that of apo-form (RMSD ∼ 4.56 Å) (Figure 3C and D). Upon cA_4_ binding, the helical insert undergoes substantial movements as observed for phosphate binding (Figure 3D). In addition, cA_4_ binding also triggers a rearrangement of the catalytic center (Figure 3E). In particular, residues Thr10 and Ser11 shift towards the dimer axis for about 5.0 Å.

**Figure 4.**
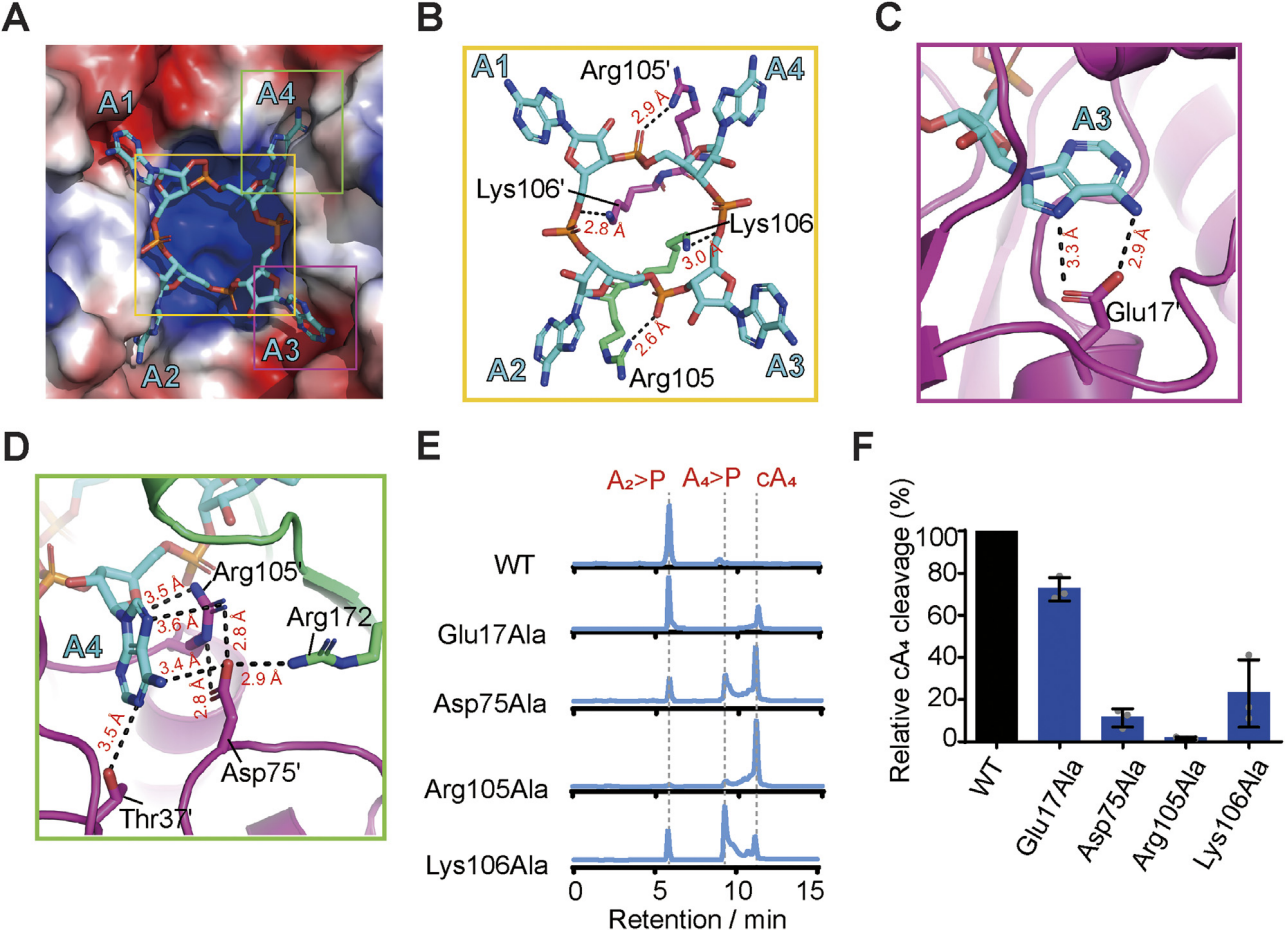
Mechanism of cA_4_ recognition by Sso2081. (**A**) Overall view of cA_4_-binding site on Sso2081 with the helical insert omitted. Sso2081 is shown in surface and cA_4_ is in stick. The surface is colored according to the electrostatic potential, with red, blue and white representing negative, positive and neutral charges, respectively. (**B**) The interactions between Sso2081 and the ribose-phosphate backbone of cA_4_. (**C**, **D**) The adenine binding sites for A3 (C) and A4 (D) of cA_4_. Dashed lines indicate hydrogen bonds. Residues involved in cA_4_ binding are shown as sticks and labeled. (**E**) Representative LC spectra of the reaction products of cA_4_ with wild type (WT) Sso2081 or its variants. Reactions were conducted at 60°C for 30 min, using 2 μM Sso2081 proteins and 40 μM synthetic cA_4_. (**F**) Quantitation of cA_4_ cleavage in (E). Values are means ± SD, *n* = 3.

The ring-shaped phosphodiester backbone of cA_4_ perfectly docks onto the prominent central basic patch, whereas the four adenine groups splay outwards and each makes contact with a binding pocket (Figure 4A). In response to cA_4_ binding, Lys106 on the top of dimeric helices (α4-α4′) from both monomers put their side chains into the ring center and point towards the two scissile phosphates (Figure 4B). In addition, Arg105 stretches its guanidinium group outside of the ring and coordinates the non-scissile phosphate (Figure 4B). The adenine bases of A1 and A3 interact mainly with the carboxylate side chain of Glu17 through two hydrogen bonds (Figure 4C and Supplementary Figure S4A), while the adenine groups of A2 and A4 are specifically recognized by the hydrogen-bond network formed between Asp75, Arg105 and Arg172 (Figure 4D and Supplementary Figure S4B). The Arg105Ala/Lys106Ala double mutation has previously been shown to abolish the catalytic activity of Sso2081 (26).Here, we found that the single mutations of Asp75, Arg105 and Lys106 greatly diminished cA_4_ cleavage (Figure 4E and F). In addition, mutation of Glu17Ala also slightly reduced the activity of Sso2081 in cA_4_ cleavage (Figure 4E and F).

**Figure 5.**
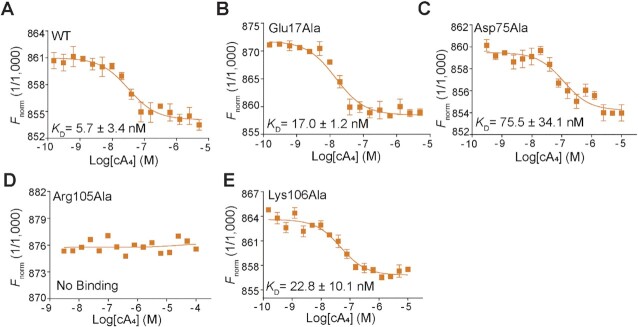
Binding isotherms for cA_4_ to Sso2081 and its variants. The binding affinities of cA_4_ with WT (**A**) and mutant (**B**–**E**) Sso2081 were evaluated by the microscale thermophoresis (MST) binding assay, using 50 nM of the labelled proteins and varying concentrations of cA_4_. Values are means ± SD, *n* = 3.

To further distinguish the contribution of these residues for binding and catalysis, we next performed a microscale thermophoresis (MST) binding assay. Sso2081 exhibited high binding affinity with cA_4_, with a *K*_D_ value of 5.7 nM (Figure 5A). Alanine substitutions of Glu17 and Lys106 caused about 3∼5-fold reduction of the binding affinity, whereas the Asp75Ala mutation resulted in ∼13-fold decrease (Figure 5B, C, E). Notably, the Arg105Ala mutation completely abolished Sso2081 binding to cA_4_ (Figure 5D). These data thus suggest that Asp75 and Arg105 make the major contributions for cA_4_ binding.

### Mechanism of cA_4_ cleavage by sso2081

**Figure 6.**
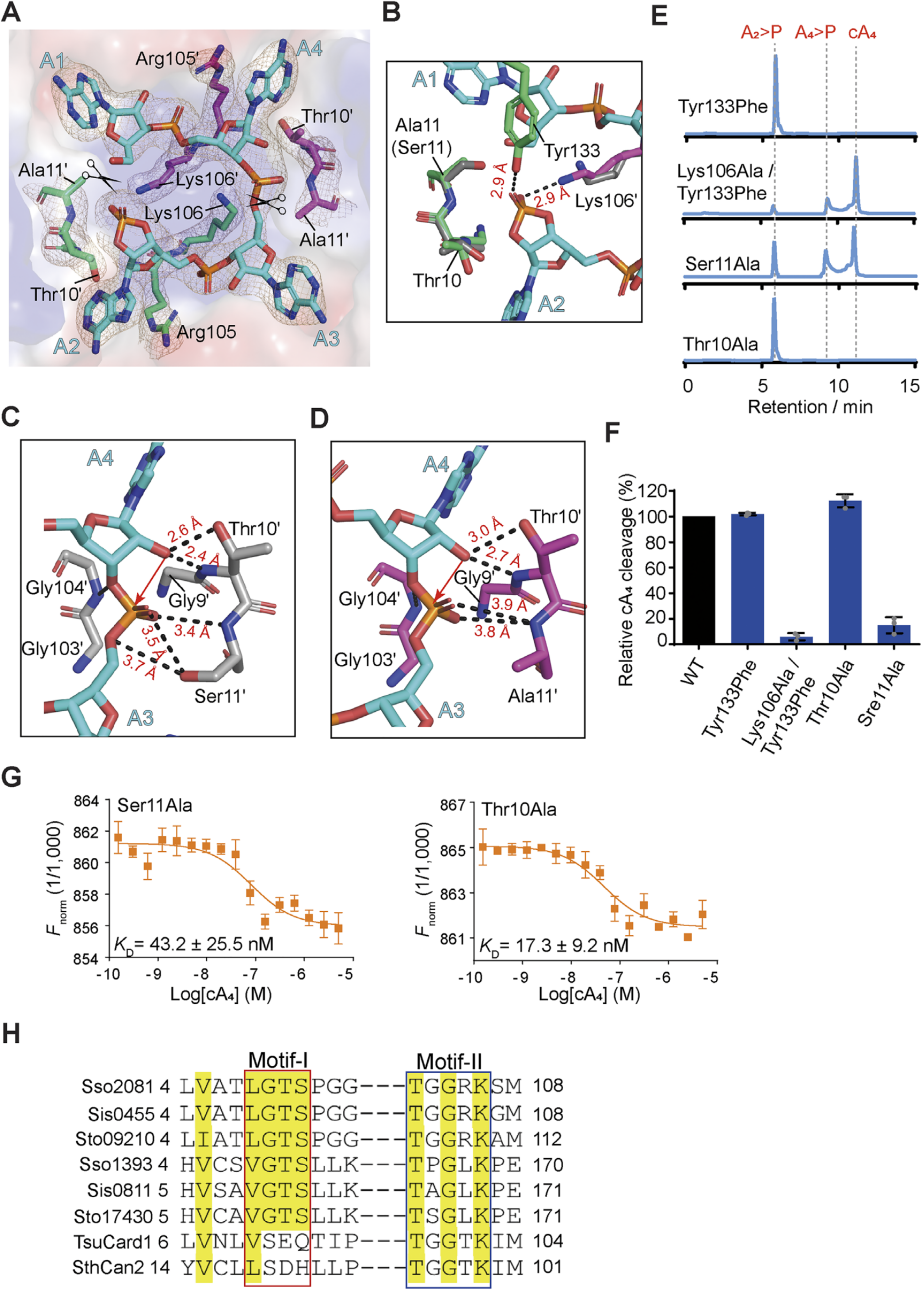
Mechanism of cA_4_ cleavage by Sso2081. (**A**) The binding site of A_4_> P in the structure of Sso2081^Ser11Ala^ /A_4_> P complex. The cA_4_ and its interacting residues are shown as sticks and overlaid with the 2Fo-Fc electron density map contoured at 1.0 σ. The cleavage site of cA_4_ is marked with a scissor symbol. (**B**) Coordination of the cleaved phosphate in the structure of Sso2081^Ser11Ala^/A_4_> P. Grey sticks indicate the conformations of key residues at the pre-cleavage state. (**C**, **D**) Close-up views of the scissile phosphate coordination in the structures of the Sso2081/cA_4_ (C) and Sso2081^Ser11Ala^/A_4_> P (D) complexes. cA_4_ is shown in cyan stick. Dashed lines indicate hydrogen bonds. Red arrow indicates nucleophilic attack. (**E**) Representative LC spectra of the reaction products of the indicated Sso2081 mutants with cA_4_. Reactions were conducted at 60°C for 30 min, using 2 μM Sso2081 proteins and 40 μM synthetic cA_4_. (**F**) Quantitation of cA_4_ cleavage in (E). Values are means ± SD, *n* = 3. (**G**) Binding isotherms for cA_4_ to Sso2081^Ser11Ala^ and Sso2081^Thr10Ala^ by the MST binding assay. Values are means ± SD, *n* = 3. (**H**) Sequence alignment between Sso2081 and its structural homologs. The conserve residues are highlighted with yellow shading. Sso, *Saccharolobus solfataricus*; Sis, *Sulfolobus islandicus*; Sto, *Sulfolobus tokodaii*; Tsu, *Treponema succinifaciens;*Sth, *Sulfobacillus thermosulfidooxidans*.

We next sought to gain further insights into the catalytic mechanism of cA_4_ cleavage by Sso2081. To this end, we determined a 2.50-Å structure of cA_4_ in complex with Sso2081^Ser11Ala^, which has previously been shown to be impaired for catalysis (26) (Table 1). The overall structure of Sso2081^Ser11Ala^ is virtually identical to that of cA_4_-bound WT Sso2081, with an RMSD of 0.56 Å (Supplementary Figure S4C–E). The electron density map of cA_4_ revealed that the phosphodiester bond connecting A1 and A2 was cleaved, whereas the phosphodiester bond at the opposing site (A3–A4) remained intact (Figure 6A). This suggests that the structure was determined at the cleavage intermediate state, and that the two active sites need not to function in a concerted manner.

In the structure of cleavage intermediate complex, the cyclic 2′,3′-phosphate of A2 as the product of first cleavage is stabilized by the side chains of Lys106′ and Tyr133 (Figure 6B). Comparing to the pre-cleavage state, the ϵ amino group of Lys106′ makes about 90° rotation and intimately contact the cleaved phosphate (Figure 6A and B), demonstrating that Lys106 may play a critical role in the stabilization of transient intermediate. In addition, the helical insert residue Tyr133, which binds the phosphate ion in the structure of Sso2081/phosphate complex, is also implicated in the cleaved phosphate coordination (Figure 6B). Although single mutation of Tyr133Phe did not result in a noticeable effect on cA_4_ cleavage, it significantly decreased the production of linear intermediate (A_4_ > P) by Sso2081^Lys106Ala^ (Figures 4E, F and 6E, F). MST binding assay showed that Sso2081^Lys106Ala/Tyr133Phe^ has comparable cA_4_ binding affinity to Sso2081^Lys106Ala^ and Sso2081^Tyr133Phe^ (Figure 5E and Supplementary Figure S4F, G). Thus, Tyr133 may play an auxiliary role in the positioning of scissile phosphates.

Similar to the phosphate ion in the Sso2081/phosphate complex, the scissile phosphate of cA_4_ in the Sso2081/ cA_4_ complex is coordinated by residues Gly9, Thr10 and Ser11, and Gly104 (Figure 6C). Substitution of Ser11 with alanine caused slight but may be critical change in the scissile phosphate coordination: the distances between the scissile phosphate and Thr10 and Ala11 (Ser11) increased for about 0.4 Å (Figure 6D). Mutation of Ser11Ala severely diminished the activity of Sso2081, whereas the replacement of Thr10 with alanine did not have a noticeable effect (Figure 6E and F), suggesting that the OH-group of Ser11 but not Thr10 is critical for scissile phosphate binding. Consistent with this, the Ser11Ala mutation led to about 8-fold decrease of Sso2081 binding to cA_4_, whereas mutation of Thr10Ala only caused ∼3-fold reduction (Figure 6G). We proposed that residues G1y9, Thr10 and Ser11 may play a fundamental role in the positioning of 2′-OH group for inline nucleophilic attack of scissile phosphate. Since we did not observe major conformational changes between the structures of Sso2081^Ser11Ala^/A_4_ > P and Sso2081/cA_4_, including the O2′-P-O5′ angles at the cleavage sites of cA_4_ (165° for WT and 173° for Ser11Ala), we propose that a subtle change in the distance between the scissile phosphate and catalytic residues might result in large difference in catalytic efficiency.

## DISCUSSION

In this study, we describe the crystal structures of the founding member of ring nuclease family Sso2081, alone, bound by phosphate ions or cA_4_ in both pre-cleavage and cleavage intermediate states. Comparing to many other ring nucleases, the most prominent structural feature of Sso2081 is in its C-terminal helical insert. In the apo-state, the helical inserts of the two monomers stand apart from each other, allowing the catalytic center wide-open for substrate access. Upon ligand binding (phosphate ion or cA_4_), the helical inserts move towards the dimer axis to completely enclose the ligands in the active site, and thereby locks the catalytic center in a closed conformation. Our structures thus reveal a gate-locking mechanism for ligand binding by the ring nuclease Sso2081. Likewise, the ring nuclease AcrIII-1 / Crn2 contains a mobile loop which undergoes a substantial movement upon cA_4_ binding (28), suggesting that this mechanism may also apply to AcrIII-1.

While our manuscript was under revision, Molina *et al.* reported the structures of a close orthologue of Sso2081 in *S. islandicus* (Sis0455) in both apo and cA_4_-bound states (33). Sis0455 shares a close structural similarity with Sso2081, with an RMSD of ∼0.8 Å (Supplementary Figure S5A). As Sso2081, Sis0455 contains a C-terminal helical insert which undergoes a similar structural arrangement upon cA_4_ binding (Supplementary Figure S5B). In addition, the key residues involving cA_4_ binding of both proteins are also very similar (Supplementary Figure S5C). However, the biochemical data suggest that there is a difference in the roles of some conserved catalytic residues. For example, mutation of the strictly conserved residue Lys106 in Sis0455 dramatically reduces cA_4_ binding (∼2000-fold) and abolishes the ring nuclease activity (33), whereas the equivalent mutation of Sso2081 only causes about 3–5-fold reduction of cA_4_ binding and results in substantial accumulation of A_4_ > P linear intermediate. Moreover, mutation of another conserved residue Ser11, which does not seem to be critical for the catalysis of Sis0455, severely diminishes the cleavage activity of Sso2081 (∼80% decrease). Thus, although Sso2081 and Sis0455 share high structural similarity, there are variations in substrate binding and catalysis.

As another important cA_4_-degrading enzyme for *S. solfataricus*, Sso1393 has been shown to be ∼10-fold less active than Sso2081 (26). Unlike Sso2081, Sso1393 does not contain the C-terminal helical insert but has an additional wHTH domain at the C-terminus, which has previously been shown to have an auto-inhibitory effect for the orthologue of Sso1393 in *S. islandicus* (Sis0811) (27). Therefore, we propose that these structural variations may lead to the difference in catalytic efficiency between Sso1393 and Sos2081.

The CARF domain-containing proteins are important components of the type III CRISPR system to combat the invasions of foreign genetic elements. CARF domains are often fused to various effectors, such as HEPN, PD-D/ExK nuclease, AAA ATPase or HTH motifs. According to the differences in structural features, association with distinct effectors, and presence or absence of the ring nuclease activity, CARF domains have been grouped into 10 major and several minor clades (45). Sequence analysis reveals that there are at least two motifs (motif-I and motif-II) critical for the ring nuclease activity of those CARFs (Figure 6H) (45). In Sso2081, the motif-I consists of residues Gly9, Thr10 and Ser11, while the motif-II is composed of residues Gly104, Arg105 and Lys106. Mechanistically, the hydrolysis of a cyclic oligoadenylate proceeds by the nucleophilic attack of the ribose 2′-OH group onto the scissile phosphate bond, producing a 2′, 3′-cyclic phosphate and a 5′-OH (26,45). Our cleavage and binding data suggest that the motif-I in Sso2081 may function mainly through the proper positioning of the 2′-OH group for inline nucleophilic attack on the scissile phosphates. In the motif-II, Arg105 makes a major contribution for substrate binding, whereas Lys106 plays an important role in the stabilization of transient intermediate.

A structural homology search with the DALI server shows that the CARF domain of Sso2081 is structurally related to that of Can2 (11,21) and Card1 (22) (Supplementary Figure S2), however, neither Can2 nor Card1 possesses cA_4_ cleavage activity. Sequence alignment shows that Can2 and Card1 do not contain the ‘GTS’ sequence in motif-I, which is important for the catalysis of Sso2081 (Figure 6H and Supplementary Figure S6). Thus, our results support the previous notion that motif-I can be a critical signature to discriminate whether a CARF domain has cOA-degrading activity or not. In addition, both proteins possess the conserved lysine (Lys99 for Can2 and Lys102 for Card1) but not the arginine in motif-II (Figure 6H and Supplementary Figure S6), implying that in these ring nucleases the conserved lysine may play a dominant role in substrate binding.

As a summary, in this work, we have determined the crystal structures of Sso2081, alone or bound to cA_4_ at both pre-cleavage and cleavage intermediate states. These structural and biochemical data provide molecular details of how cA_4_ is specifically recognized and cleaved by Sso2081, and suggest a gate-locking mechanism for ligand binding. Furthermore, the key residues and motifs identified herein for substrate binding and catalysis provide a new insight to discriminate between cOA-degrading and -nondegrading CARF domain-containing proteins.

## DATA AVAILABILITY

Atomic coordinates and structure factors for the reported crystal structures have been deposited with the Protein Data bank under accession number 8HTW (Sso2081^Tyr133Phe^), 7YHL (Sso2081/phosphate), 7YGH (Sso2081/cA_4_), 7YGL (Sso2081^Ser11Ala^/cA_4_ > P).

## Supplementary Material

gkad101_Supplemental_File
