# Supplementary material for: Molecular basis of stepwise cyclic tetra-adenylate cleavage by the type III CRISPR ring nuclease Crn1/Sso2081
Source: Nucleic Acids Res. 2023 Feb 20;51(5):2485–95. doi: 10.1093/nar/gkad101 (PMC10018336; doi:10.1093/nar/gkad101)
Supplement: gkad101_Supplemental_File [file gkad101_supplemental_file.pdf]

Molecular basis of stepwise cyclic tetra-adenylate cleavage by the type III CRISPR ring nuclease Crn1/Sso2081

Liyang Du[1], Danping Zhang[1], Zhipu Luo[2,*] & Zhonghui Lin[1,*]

[1]College of Chemistry, Fuzhou University, Fuzhou 350108, China.

[2]Institute of Molecular Enzymology, School of Biology and Basic Medical Sciences, Soochow University, Suzhou, China.

[*]Correspondence: E-mail: luozhipu@suda.edu.cn (Z. Luo); zhonghui.lin@fzu.edu.cn (Z. Lin)

SUPPLEMENTARY FIGURES

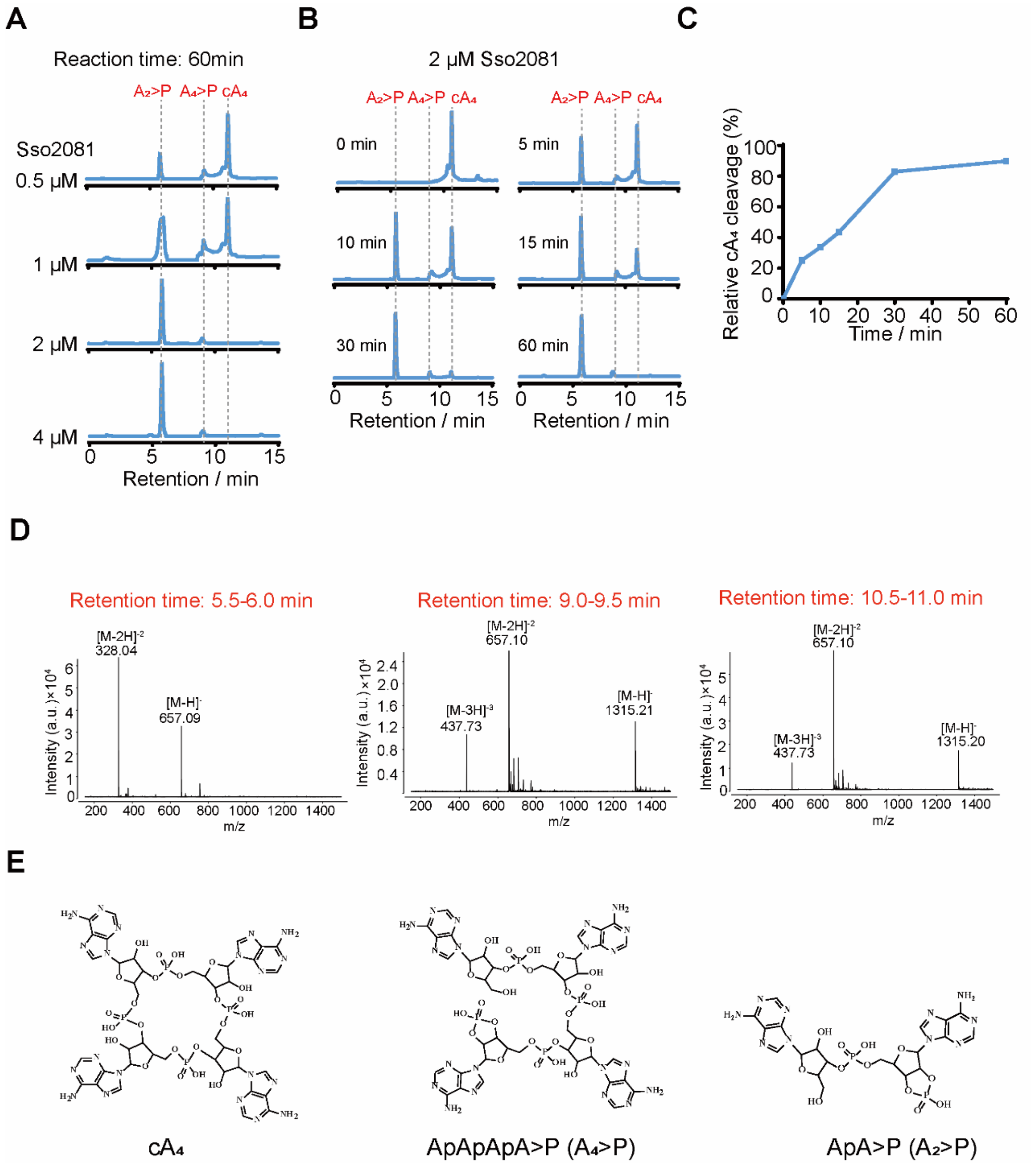

Supplementary Fig. S1 LC and MS analyses of cA_4 cleavage by Sso2081. (A) LC spectra of the reaction products of cA_4 with increasing concentrations of Sso2081. Reactions were conducted at 60 °C for 60 min. **(B)** LC spectra of the reaction products of cA_4 with 2 μM Sso2081 at 60 °C for 0 ~ 60 min. **(C)** The kinetic plot of cA_4 cleavage in (B). **(D)** Mass spectra of the samples eluted from HPLC column at indicated retention

times. Retention time 5.5-6.0: *m/z* 657.09 for $A_2>P^{-1}$ ($ApA>P^{-1}$); *m/z* 328.04 for $A_2>P^{-2}$; Retention time 9.0-9.5 min: *m/z* 1315.21 for $A_4>P^{-1}$ ($ApApApA>P^{-1}$); 657.10 for $A_4>P^{-2}$; 437.73 for $A_4>P^{-3}$; Retention time 10.5-11.0: *m/z* 1315.20 for cA_4^{-1}; *m/z* 657.10 for cA_4^{-2}; *m/z* 437.73 for cA_4^{-3}. **(E)** The chemical structures of cA_4 and the cleavage products.

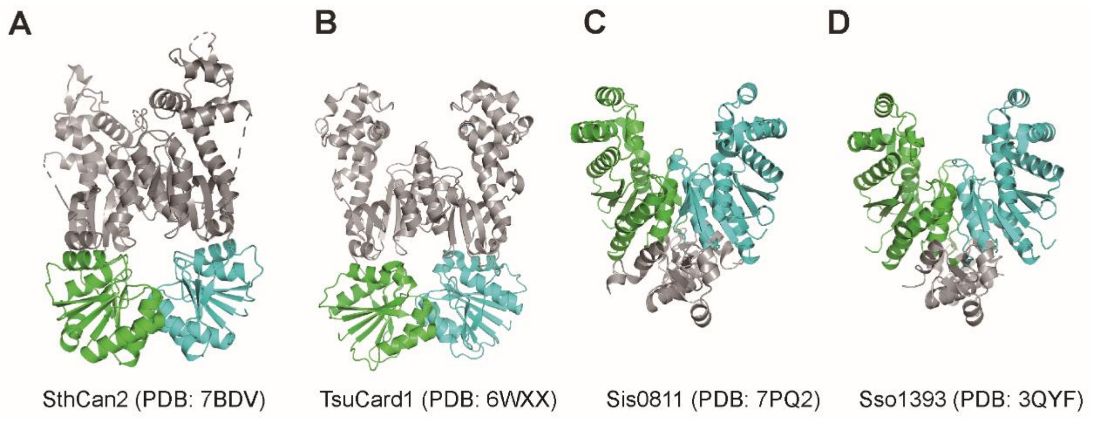

Supplementary Fig. S2 Comparison of the structures between various CARF domain-containing proteins. A-D, Structures SthCan2, TsuCard1, Sis0811 and Sso1393. The structures are shown in cartoon representation. CARF domains are highlighted in green / cyan colors. Sis, *Sulfolobus islandicus*; Sso, *Sulfolobus solfataricus*; Sth, *Sulfobacillus thermosulfidooxidans*; Tsu, *Treponema succinifaciens*.

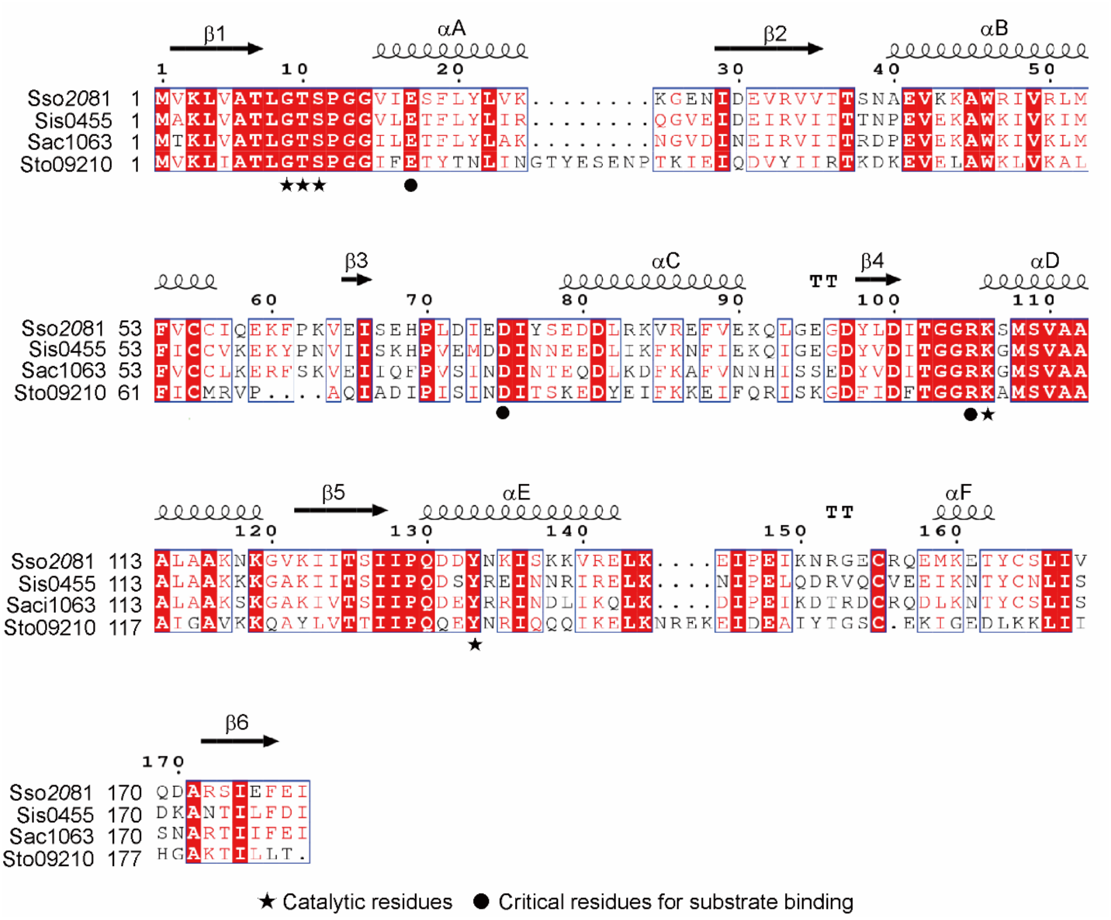

Supplementary Fig. S3 Sequence alignment of Sso2081 with its structural homologs. The alignment is generated using the online ESPript 3.0 server. Secondary structural elements of Sso2081 are indicated above the sequences. Abbreviations: Sso, *Saccharolobus solfataricus*; Sis, *Sulfolobus islandicus*; Sac, *Sulfolobus acidocaldarius*. Sto, *Sulfolobus tokodaii*;

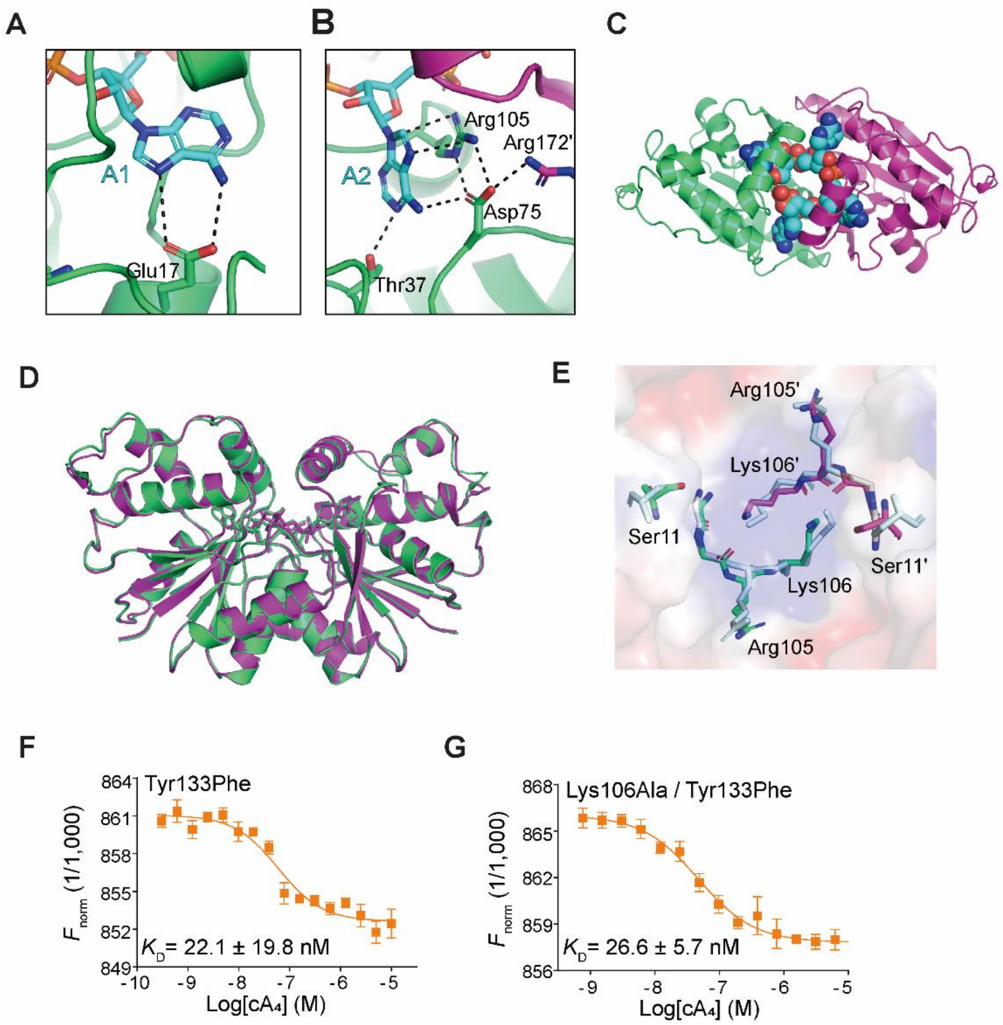

Supplementary Fig. S4 Structural comparison between cA$_4$- and A$_4$>P-bound Sso2081. (A, B) The adenine binding sites for A1 (A) and A2 (B) of cA$_4$ in the complex of Sso2081/cA$_4$. **(C)** Top-view of the structure of Sso2081/A$_4$>P complex. Sso2081 is shown in cartoon representation and cA$_4$ is in sphere. **(D)** Superposition of the structures of cA$_4$- (green) and A$_4$>P- (magenta) bound Sso2081. **(E)** Local conformational changes in the active site of Sso2081 between phosphate- (light blue) and cA$_4$- (green and magenta) bound Sso2081. The structure is overlaid with 80% transparent surface of the active site of cA$_4$-bound Sso2081. **(F, G)** Binding isotherms

for cA_4 to $Sso2081^{Tyr133Phe}$ (F) and $Sso2081^{Lys106Ala/Tyr133Phe}$ (G) by the MST binding assay. Values are means ± SD, n = 3.

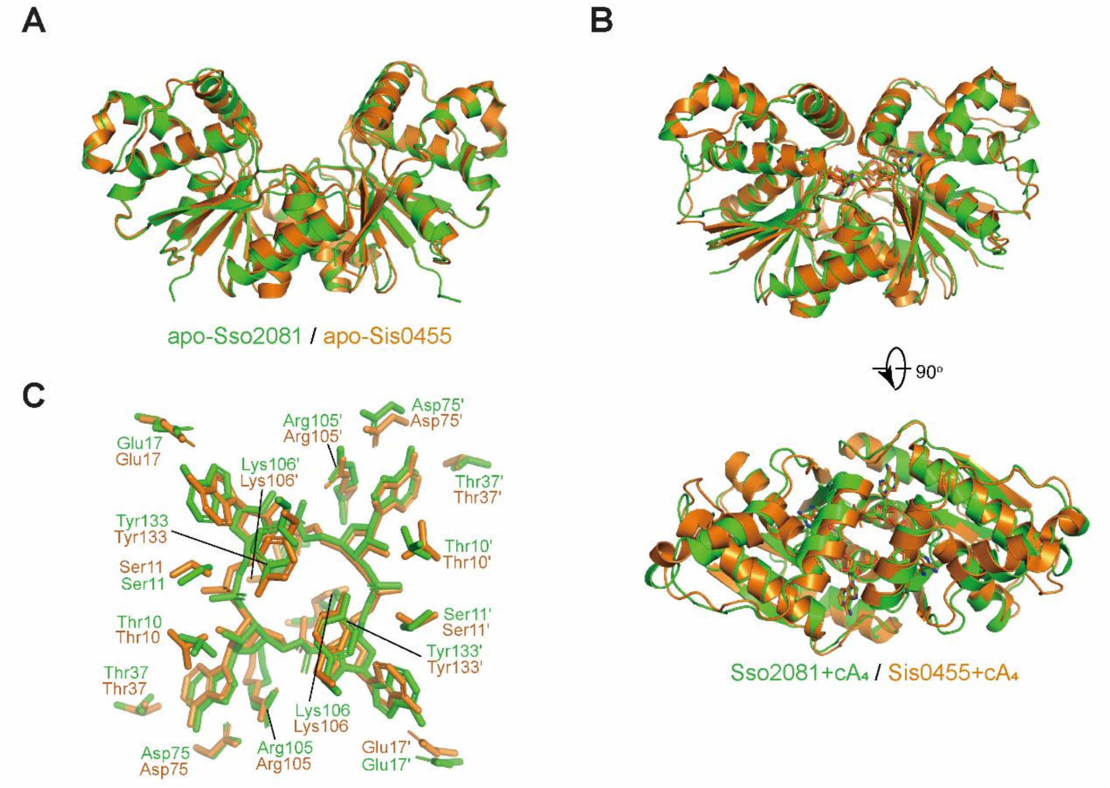

Supplementary Fig. S5 Structural comparison between Sso2081 and Sis0455. (A) Structural comparison between apo-Sso2081 (green) and apo-Sis0455 (orange, PDB: 7Z56) from side view. **(B)** Structural comparison between cA_4-bound Sso2081 (green) and Sis0455 (orange, PDB: 7Z55) from both side (top panel) and top (bottom panel) views. **(C)** Comparison of the cA_4-binding residues between Sso2081 and Sis0455.

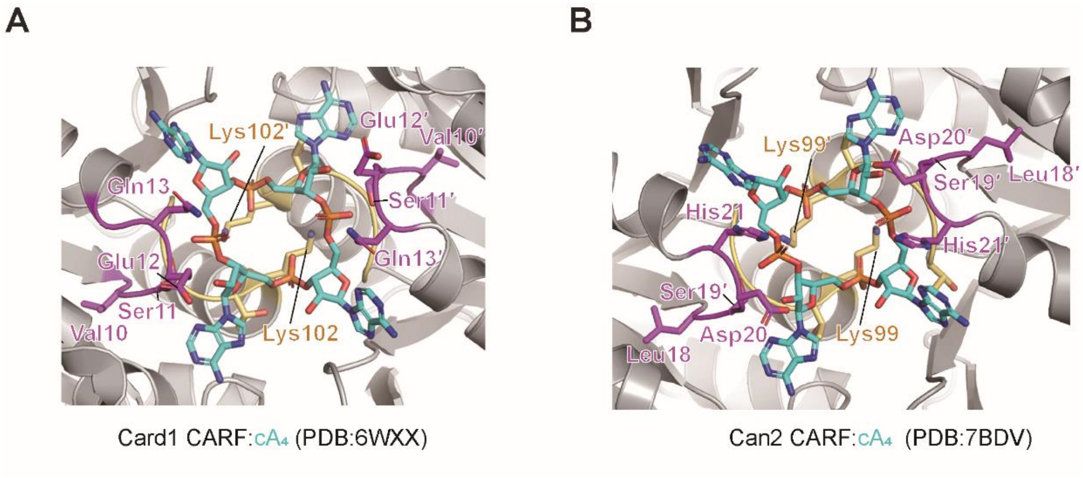

Supplementary Fig. S6 The cA_4 binding site of Card1 and Can2. (A) The cA_4 binding site in the structure of TsuCard1 in complex with cA_4 (PDB: 6WXX). **(B)** The cA_4 binding site of Can2 in the structure of SthCan2 in complex with cA_4 (PDB: 7BDV). cA_4 is shown in cyan stick. Key residues in motif-I and motif-II are labeled and shown in magenta and yellow sticks, respectively.
